# From Gene Trees to Organismal Phylogeny in Prokaryotes:The Case of the γ-Proteobacteria

**DOI:** 10.1371/journal.pbio.0000019

**Published:** 2003-09-15

**Authors:** Emmanuelle Lerat, Vincent Daubin, Nancy A Moran

**Affiliations:** **1**Department of Ecology and Evolutionary Biology, University of ArizonaTucson, ArizonaUnited States of America; **2**Department of Biochemistry and Molecular Biophysics, University of ArizonaTucson, ArizonaUnited States of America

## Abstract

The rapid increase in published genomic sequences for bacteria presents the first opportunity to reconstruct evolutionary events on the scale of entire genomes. However, extensive lateral gene transfer (LGT) may thwart this goal by preventing the establishment of organismal relationships based on individual gene phylogenies. The group for which cases of LGT are most frequently documented and for which the greatest density of complete genome sequences is available is the γ-Proteobacteria, an ecologically diverse and ancient group including free-living species as well as pathogens and intracellular symbionts of plants and animals. We propose an approach to multigene phylogeny using complete genomes and apply it to the case of the γ-Proteobacteria. We first applied stringent criteria to identify a set of likely gene orthologs and then tested the compatibilities of the resulting protein alignments with several phylogenetic hypotheses. Our results demonstrate phylogenetic concordance among virtually all (203 of 205) of the selected gene families, with each of the exceptions consistent with a single LGT event. The concatenated sequences of the concordant families yield a fully resolved phylogeny. This topology also received strong support in analyses aimed at excluding effects of heterogeneity in nucleotide base composition across lineages. Our analysis indicates that single-copy orthologous genes are resistant to horizontal transfer, even in ancient bacterial groups subject to high rates of LGT. This gene set can be identified and used to yield robust hypotheses for organismal phylogenies, thus establishing a foundation for reconstructing the evolutionary transitions, such as gene transfer, that underlie diversity in genome content and organization.

## Abstract 2

The study demonstrates that single-copy orthologous genes are resistant to horizontal transfer and can be used to generate robust hypotheses for organismal phylogenies

## Introduction

The availability of complete sequences of genomes for clusters of related organisms presents the first opportunity to reconstruct events of genomic evolution. By comparing related genomes and inferring ancestral ones, we can identify events, such as specific chromosomal rearrangements, gene acquisitions, duplications, and deletions, that have produced the observed diversity in genome content and organization. The Bacteria offer the most immediate opportunities for such reconstruction, because many clusters of related genomes are now available and because the genomes are small and contain relatively little repetitive sequence, reducing computational complexity. Among bacterial groups, the γ-Proteobacteria presents the most intensively studied and sequenced cluster of genomes with varying degrees of relatedness.

Intertwined with the problem of reconstructing genomic change is the problem of inferring phylogeny. Evading this issue is particularly difficult in the Bacteria. First, using complete genomes to obtain a robust phylogeny for all bacteria has presented problems due to the age of the group and the resulting loss of phylogenetic signal. Furthermore, lateral gene transfer (LGT) occurs in bacteria and has been claimed to be rampant for all classes of genes, potentially resulting in a diversity of phylogenetic histories across genes and complicating, or completely defeating, attempts to reconstruct bacterial evolution at both deep and more recent evolutionary depths (Doolittle 1999; Nesbø et al. 2001; Gogarten et al. 2002; Wolf et al. 2002; Zhaxybayeva and Gogarten 2002). Although the existence of substantial levels of LGT in bacterial genomes is not disputed, the existence of a core of genes resistant to LGT has been proposed (Jain et al. 1999) and has received some support from recent studies using relatively intensive taxon sampling (Brochier et al. 2002; Daubin et al. 2002).

For purposes of reconstructing genomic change, what we seek is the organismal phylogeny—that is, the topology that traces the history of the replicating cell lineages that transmit genes and genomes to successive generations. The organismal phylogeny provides the backdrop against which events of genomic change, including LGT, have occurred. High incidence of LGT may cause the organismal phylogeny to be elusive, because we do not know which genes represent the true history of the cell lineages.

The gene most used for reconstructing organismal phylogeny is the small subunit ribosomal RNA (SSU rRNA), which has been argued to rarely undergo transfer among genomes (Woese 1987; Jain et al. 1999). But even this gene may undergo occasional LGT or recombination (Ueda et al. 1999; Yap et al. 1999). Furthermore, by itself, it provides insufficient information to resolve phylogenies, particularly for cases of heterogeneous rates and patterns of substitution. Thus, building conclusions about organismal phylogeny on the basis of SSU rRNA alone is unsatisfactory. The availability of complete genome sequences presents us with the potential to exploit the much greater set of genes that are expected to share the same history of transmission along the branches of the organismal phylogeny. A robust phylogeny based on more sequences could then be used to reconstruct genome-scale events, including LGT and rearrangements. But, while complete genome sequences have enormous potential for addressing phylogenetic issues, their utility for reconstructing bacterial phylogeny is initially quite limited due to the requirement of thorough taxon sampling within a clade for accurate reconstruction of phylogenies (Zwickl and Hillis 2002; Hillis et al. 2003). Only now, with the continuing increase in numbers of fully sequenced bacterial species, is it becoming possible to obtain sufficiently dense taxon sampling to exploit the large amount of genomic sequence data for the purpose of phylogeny reconstruction.

We have chosen one group of Bacteria, the γ-Proteobacteria, to address the problem of whether complete genome sequences can be used for robust reconstruction of the organismal phylogeny, despite high levels of LGT. The γ-Proteobacteria, distinguished on the basis of sequence signatures and structural differences in the SSU rRNA (Woese 1987), is an ideal choice for this purpose. This group represents a model of bacterial diversification and includes free-living and commensal species, intracellular symbionts, and plant and animal pathogens. The sequence divergence of certain of its members (Clark et al. 1999) suggests an age of at least 500 million years. At the same time, members are sufficiently closely related to enable us to reduce the problem of lack of phylogenetic signal and to identify a large set of unambiguous orthologs. Currently, the γ-Proteobacteria contains the highest density of fully sequenced genomes, including those of species (Escherichia coli and Salmonella sp.) for which knowledge of gene function is more complete than for any other cellular organisms. The potential obstacles to phylogenetic inference that are found across the Bacteria are certainly present in the γ-Proteobacteria. In particular, LGT is known to be extensive in this group, based on studies of genome composition (Lawrence and Ochman 1997; Parkhill et al. 2000, 2001; Stover et al. 2000). Symbiotic lineages present particular issues for phylogeny reconstruction owing to huge losses of genes (Shigenobu et al. 2000; Akman et al. 2002), accelerated sequence evolution, and shifts in base composition (Moran 1996). These features create phylogenetic artifacts and make the use of additional data from genome sequences particularly desirable.

Here we aim to use complete genome sequences to reconstruct the organismal phylogeny for the γ-Proteobacteria by first selecting a set of probable ortholog families and then determining whether most agree on a common topology. A major implication of our results is that the replacement of single-copy orthologous genes is extremely rare, even within phyla. Instead, LGT most often involves uptake of genes assuming functions that are not represented in the recipient and arriving from distantly related bacteria or from phage (Daubin et al. 2003a; Pedulla et al. 2003). A consequence is that most single-copy orthologous genes show broad phylogenetic agreement that reflects the organismal relationships and that provides a foundation for reconstructing events of genome evolution.

## Results

### Gene Families and Identification of Orthologous Genes

**Figure 1:**
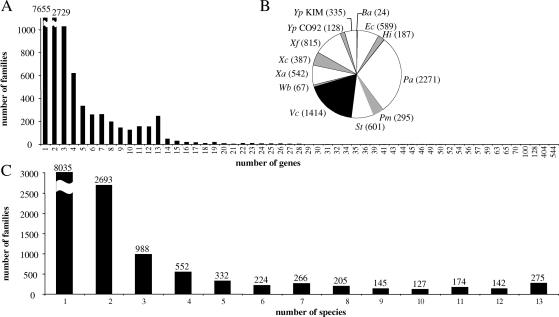
Number of Genes and Species within the Gene Families (A) Distribution of the number of genes contained in the homolog families. (B) Number of orphan genes in each species in parentheses. Abbreviations: Ba, Buchnera aphidicola; Ec, Escherichia coli; Hi, Haemophilus influenzae; Pa, Pseudomonas aeruginosa; Pm, Pasteurella multocida; St, Salmonella typhimurium; Vc, Vibrio cholerae; Wb, Wigglesworthia brevipalpis; Xa, Xanthomonas axonopodis; Xc, Xanthomonas campestris; Xf, Xylella fastidiosa; Yp CO92, Yersinia pestis CO_92; Yp KIM, Yersinia pestis KIM. (C) Distribution of the number of species contained in the homolog families.

The proteins of 13 complete γ-proteobacterial genomes were classified into an initial set of 14,158 homolog families, using the procedures described in Materials and Methods. Figure 1A shows the distribution of the number of genes per family. A majority (7,655) of the families contain only one gene. As the criteria we applied for grouping genes into families are stringent, this number is expected to exceed the number of real orphan genes; indeed, annotations for many of these genes do claim homology with other genes in the included genomes. As a result, values for most of the genomes (Figure 1B) are higher than the number of genes annotated as orphans; for example, the number of this type of gene identified for Buchnera was 24, but the annotation indicates only four genes unique to this species (Shigenobu et al. 2000). Moreover, our comparison was made only within this group of 13 bacteria, and some single-gene families may have homologs in other, more distant bacterial species. Pseudomonas aeruginosa yielded the highest number of unique genes, which represent nearly 41% of proteins of this genome. This is congruent with the result obtained during the original annotation of this genome: the authors were unable to identify relatives for 32% of the ORFs (Stover et al. 2000).

At the other extreme, some families group large numbers of genes. The largest family contains 544 genes and corresponds to the ABC transporter family, known to be the largest protein family (Tatusov et al. 1996; Tomii and Kanehisa 1998). The second-largest family, with 404 genes, corresponds to the histidine kinase response regulators (Wolanin et al. 2002).

Figure 1C shows the distribution of number of species per gene family. Note that a large majority of families group only one or two species (8,035 and 2,693 families, respectively). In the families comprising only one species, Pseudomonas and Vibrio are heavily represented, with 2,397 and 1,474 families, respectively. The families containing two species often group two closely related genomes, such as the two Xanthomonas, the two Yersinia, Escherichia and Salmonella, and Haemophilus and Pasteurella.

A total of 275 families are represented in all 13 species. Among these, 205 contain exactly one gene per species. We consider these 205 genes to represent likely orthologs and, consequently, to be good candidates for use in inferring the organismal phylogeny and the extent of LGT.

### The Extent of Conflict among Gene Families

**Figure 2:**
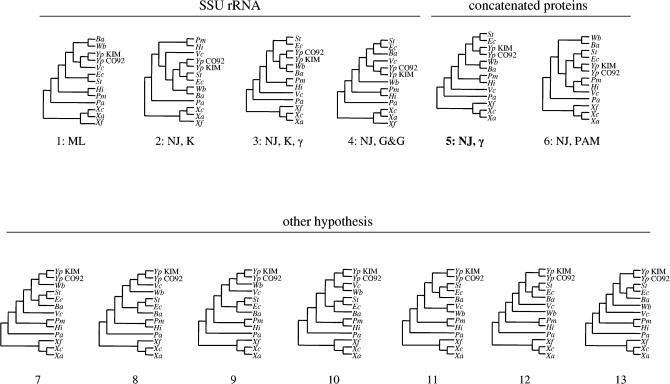
The 13 Candidate Topologies Topologies 1–4 correspond to tree reconstructions based on SSU rRNA. Topologies 5 and 6 correspond to the trees based on the concatenation of the proteins. Topologies 7–13 correspond to additional topologies constructed to test the sister relationship of the two symbiont species. Species abbreviations as in Figure 1. Abbreviations: ML, maximum likelihood; NJ, neighbor joining; K, Kimura distance; G&G, Galtier and Gouy distance; γ, gamma-based method for correcting the rate heterogeneity among sites. The position of the root corresponds to the one obtained repeatedly using SSU rRNA.

We constructed trees based on several combinations of data and methods (see Materials and Methods), with the aim of generating a set of candidate topologies for the organismal phylogeny. These seven analyses produced a total of six topologies (numbered 1–6 in Figure 2). (The identical topology was obtained for the consensus tree and the tree based on the protein concatenation using the neighbor-joining [NJ] method and the γ-based method for correcting the rate heterogeneity among sites.) The trees differ, in particular, with regard to the positions of Wigglesworthia, Buchnera, and Vibrio. All topologies, except number 4 (that one resulting from the Galtier and Gouy distance method with the SSU rRNA), tend to place Wigglesworthia and Buchnera as sister taxa (Figure 2). The sister relationship of Wigglesworthia and Buchnera was of particular interest because it would suggest a shared origin of symbiosis in an ancestor of these two species. Thus, we tested seven additional topologies (numbered 7–13 on Figure 2) that did not place these two species as sister taxa, but that otherwise resembled topologies 1–6.

**Figure 3:**
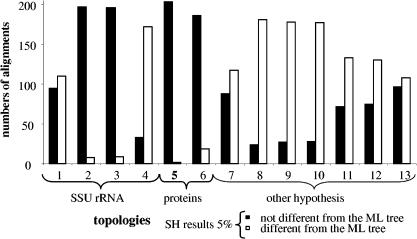
Result of the SH Test The graph shows the number of alignments accepting or rejecting each topology. The “Other Topologies” are those built to test the sister relationship of Wigglesworthia and Buchnera. The “Proteins” topologies are those obtained using both the protein concatenation and the consensus of trees from all 205 alignments. The “SSU rRNA” topologies were obtained using the SSU rRNA sequences with different methods.

For each alignment, we tested the likelihood of the 13 topologies against the maximum-likelihood (ML) topology, using the Shimodaira–Hasegawa (SH) test, as recommended by Goldman et al. (2000). The question asked here was whether the tested topologies could be considered equally good explanations of the data. Figure 3 shows the result of this test. One topology (number 5) is in agreement with 203 of 205 alignments (Figure 3). Three other slightly different topologies can be considered nearly as good on the basis of agreement with a large majority of alignments (for topologies 2, 3, and 6 agreement was with 197, 196, and 186 alignments, respectively; Figure 3).

### Cases of Lateral Transfer

**Figure 4:**
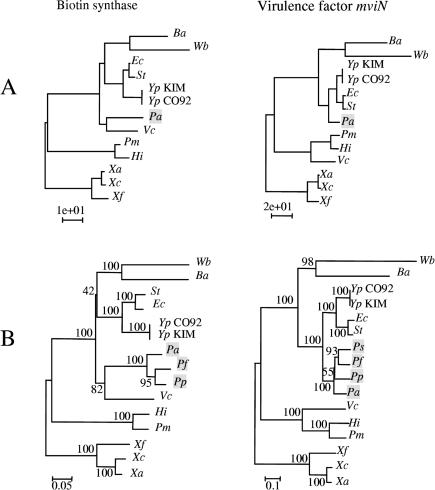
Phylogenies for the Laterally Transferred Genes (A) ML trees obtained for BioB (left) and MviN (right). (B) NJ trees obtained for BioB (left) and MviN (right). Abbreviations: Pf, Pseudomonas fluorescens; Pp, Pseudomonas putida; Ps, Pseudomonas syringae. Other species abbreviations as in Figure 1.

Of the 205 alignments, two were found to strongly reject the most accepted tree (topology 5) as well as all other topologies tested. We have investigated these two genes to determine whether the incongruence is likely to result from LGT. The proteins correspond to biotin synthase (BioB) and to the virulence factor MviN. The trees obtained using ML for each alignment are shown in Figure 4A. In both cases, the position of Pseudomonas conflicts with all widely supported topologies; it is placed as sister-group to Vibrio (BioB) or as sister-group to the enterics Escherichia, Salmonella, and Yersinia (MviN). Although initial examination of the topologies obtained from these genes suggests more than a single LGT (comparing trees of Figure 4A to topology 5 of Figure 2), the hypothesis of a single transfer in an ancestor of Pseudomonas could not be rejected for either gene based on the results of the SH test after removal of Pseudomonas from the alignments and from topology 5 and other widely supported phylogenies. The implication is that a single transfer event in an ancestor of Pseudomonas is sufficient to explain the conflict of *bioB* and *mviN* with trees derived from other genes. In addition, we searched GenBank for homologous genes in other species of Pseudomonas and built trees using NJ and the Poisson correction (Figure 4B). In each case, Pseudomonas species are grouped and display the same position as in the trees, based only on the 13 sequenced genomes (Figure 4A). Moreover, the bootstrap support was high for the grouping of Pseudomonas with Vibrio in the BioB tree and for the grouping of Pseudomonas with the enteric bacteria in the MviN tree. Thus, the phylogeny of each of these two genes can be explained as the result of a single LGT event, from different donors within the γ-Proteobacteria to a shared ancestor of these Pseudomonas species.

In Escherichia, Vibrio, Salmonella, Yersinia, and Pseudomonas, *bioB* is flanked by *bioF*, also involved in biotin biosynthesis. To determine whether *bioF* could have been transferred with the *bioB* gene, we built a tree based on the protein translation of *bioF* using all species except Buchnera, Haemophilus, and Pasteurella, which lack this gene. The tree obtained did not show any unexpected position of Pseudomonas, indicating that only *bioB* has been horizontally transmitted. A possible explanation, consistent with the flanking position of *bioB* and *bioF* in the Pseudomonas genome, is that the original *bioB* gene was replaced through homologous recombination in a common ancestor of the included Pseudomonas species. Similar comparisons for *mviN* did not illuminate its history in Pseudomonas, as the flanking genes differed from those in other species. The observations for *mviN* are consistent with a transfer event from an enteric species to a new genomic position in a Pseudomonas ancestor.

### Robustness of the Inferred Organismal Phylogeny

**Figure 5:**
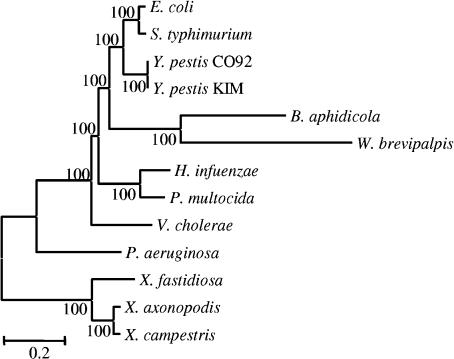
Tree Based on the Concatenation of the 205 Proteins (NJ) The topology shown agrees with almost all individual gene alignments (topology 5 of Figure 2). The same tree is obtained after removing the two genes showing evidence for LGT. The position of the root corresponds to the one obtained repeatedly using SSU rRNA.

The general lack of conflict observed among the 203 remaining families was not due to the absence of phylogenetic signal in the gene alignments because most genes did conflict with several other topologies (see Figure 3). We interpreted this congruence as a reflection of shared history and a lack of LGT. Therefore, we chose these genes as the basis for inferring the true organismal phylogeny for these 13 species. The resulting tree was the same as that for the concatenation of all of the 205 genes and for the consensus of the trees obtained from all protein families (topology 5 in Figure 2 and tree presented in Figure 5). It differed only slightly from other tested topologies (see Figure 2) that also are not rejected by many individual alignments (see Figure 3). Finally, an SH test performed using the complete concatenated alignment shows that this topology is significantly more likely than all alternative hypotheses.

All topologies separating Wigglesworthia and Buchnera were rejected by the majority of the alignments. In the best-supported topology (see Figure 5), Wigglesworthia and Buchnera are grouped and comprise the sister-group to the enteric bacteria Yersinia, Salmonella, and Escherichia. Previously published phylogenies, based on SSU rRNA, gave conflicting results for the positions of these symbionts, sometimes placing Buchnera as sister-group to Escherichia and Salmonella (van Ham et al. 1997; Spaulding and von Dohlen 1998; Moya et al. 2002). Because the genomes of these endosymbionts present a strong bias toward A+T relative to other genomes in the set, their grouping could reflect convergence at some nucleotide sites. This convergence could affect both the SSU rRNA, which is enriched in A+T (Moran 1996), and also the protein sequences, which are enriched in amino acids with A+T-rich codon families (Clark et al. 1999). To test this hypothesis, we removed from the alignment of the protein concatenation all sites at which Buchnera and Wigglesworthia contain amino acids encoded by A+T-rich codons (phenylalanine, tyrosine, methione, isoleucine, asparagine, and lysine) (Singer and Hickey 2000). Using the resulting alignment (about 30,000 residues), we have reconstructed two trees, one with the NJ method and the polyacid-modified (PAM) matrix; the other with the NJ method and the γ-based method for correcting the rate heterogeneity among sites and bootstrap. The trees obtained (data not shown) were identical and gave strong support to the grouping of Buchnera and Wigglesworthia and to their position as the sister-group of enteric bacteria (Escherichia, Salmonella, and Yersinia). Thus, this grouping is probably not an artifact of the biased composition of the endosymbiont genomes.

## Discussion

The most striking result is the almost complete lack of conflict among the set of genes selected as likely orthologs. Only two of 205 ortholog families showed such disagreement, both involving the P. aeruginosa genome. Because the γ-Proteobacteria has been the bacterial group most often cited as showing high rates of LGT, this finding is unexpected. However, we note that the evidence for LGT from sequence features and comparisons of genome content (Lawrence and Ochman 1997; Ochman and Jones 2000; Parkhill et al. 2001; Perna et al. 2001; Daubin et al. 2003a) primarily implicate genes that are absent from related bacteria; such genes would not have been retained in our set of putative orthologs. Furthermore, such genes are not candidates for phylogeny reconstruction since they are missing from most taxa. We also eliminated the large set of homolog families present as more than one sequence within even one of the genomes. If families containing paralogs show relatively high susceptibility to LGT, the proportion of genes undergoing LGT would be underestimated by considering only the set with one sequence per genome. Our aim was to locate a set of genes giving strong and consistent signal regarding the organismal phylogeny, and our results do not imply a lack of LGT in genes other than the widespread, single-copy orthologs that we selected. By streamlining the dataset for our primary goal, we have excluded genes that undergo more frequent transfer.

Phylogenetic evidence for LGT mostly involves transfer between distantly related organisms (Nelson et al. 1999; Brown et al. 2001; Brown 2003; Xie et al. 2003), and most clear-cut cases involve genes that are sporadically distributed (e.g., Parkhill et al. 2000; Singer and Hickey 2000) and thus excluded from our selection of families. The selected set includes genes that are distributed across a wide set of bacteria and includes about 100 universally distributed genes, such as those encoding ribosomal proteins, DNA polymerase subunits, and transfer RNA synthetases (Table S1). Thus, if LGT is affecting most categories of genes, it should be detectable in our set, resulting in discordance of phylogenies whether it occurs between related genomes (within the γ-Proteobacteria) or between very dissimilar genomes. Such discordance was extremely rare, affecting only two (1%) of our families.

Several previous studies have provided evidence that a core of genes may resist LGT and give a consistent phylogenetic signal (Jain et al. 1999; Brochier et al. 2002; Daubin et al. 2002). However, the same studies have noted high incidence of genes showing incongruence, and, because they involved deeper trees and incorporated a much less dense sampling of genes or of taxa, this incongruence has not been firmly identified as due to LGT or to phylogenetic artifacts. Furthermore, recent analyses based on other sets of taxa have led to the proposal that all sets of genes, including orthologous genes, are subject to high rates of LGT (Nesbø et al. 2001; Gogarten et al. 2002; Zhaxybayeva and Gogarten 2002), thereby casting doubt on the idea that we can identify a core set of orthologs that reflect the organismal phylogeny. Our analysis indicates that LGT is unusual for single-copy orthologous genes; that is, a gene copy from one species usually does not replace its ortholog in another species. The apparent discrepancy is not due to a relative lack of LGT in this particular group of bacteria, which is known to acquire foreign genes frequently (Lawrence and Ochman 1997; Parkhill et al. 2000; Perna et al. 2001). More likely explanations are that (1) our criterion for orthology was more stringent in ruling out undetected paralogy; (2) the use of quartet phylogenies (Zhaxybayeva and Gogarten 2002) can be misleading owing to artifacts linked to taxon sampling (Zwickl and Hillis 2002); and (3) our focus on a relatively closely related group of bacteria minimizes the problem of loss of phylogenetic signal and reconstruction artifacts in deep divergences. This result thus provides further evidence that, though bacterial genomes constantly acquire and lose significant amounts of DNA, the incidence of LGT for widespread orthologous genes is relatively low (Daubin et al. 2003b). Although we have likely excluded many actual orthologs, the set of retained genes provides a dataset that is sufficiently informative to give a highly resolved and well-supported phylogeny for these taxa.

This study thus defines a minimal core of genes that show both wide representation and congruent phylogenetic signal in γ-Proteobacteria. We note that this core includes numerous genes in both “informational” and “operational” functional categories (Table S1); thus, our results do not fit closely with the “complexity hypothesis,” that only informational genes avoid LGT (Jain et al. 1999), although they do not exclude such a trend. Our set of 203 genes should not be considered as representative of all genes resisting LGT, since we did not explore the other gene families. The main functional feature distinguishing the set is likely to be essentiality, owing to the requirement of presence in all 13 genomes, including the reduced symbiont genomes. For the goal of selecting genes that reflect organismal phylogeny through vertical descent, our criteria (single copy and ubiquitous) appear to be more reliable than criteria based on functional information (informational genes, translational genes, etc.). Indeed, cases of LGT are known for informational genes (e.g., Brochier et al. 2000).

One possible explanation for the lack of observed events of orthologous replacement might be that these are sufficiently rare that significant frequencies are encountered only when considering deeper phylogenetic levels. However, the group studied here, though recent enough to allow accurate phylogenetic reconstruction, is old. Indeed, the divergence of different Buchnera species has been dated to approximately 200 million years based on the host fossil record (Clark et al. 1999), and the clade we have studied must be much more ancient. A conservative molecular clock estimate, based on rRNA and dating the divergence of Escherichia and Buchnera at 200 million years, places the origin of the group at more than 500 million years (calculations not shown). Thus, our finding that very few orthologs have been exchanged within the group and that none show evidence of having been imported from other bacterial lineages is relevant for the understanding of long-term bacterial evolution.

It has been proposed that LGT may be more frequent within clusters of related bacteria and even that phylogenetic groupings, such as the γ-Proteobacteria, may reflect boundaries to LGT rather than recent shared ancestry of lineages (Gogarten et al. 2002). Such a model, which is consistent with apparent concordance among ortholog families in studies with poor taxon sampling but predicts rampant discordance within a well-sampled bacterial cluster, is strongly rejected by our results. Our findings favor the view that the cohesion of major phylogenetic groups within the Bacteria is due to vertical transmission and common ancestry rather than to preferential lateral transfer of genes. However, the results presented here do not eliminate the possibility of nonrandom patterns of LGT for gene families that are more sporadically distributed.

A robust phylogenetic framework for the organismal lineages provides the foundation for reconstructing the events of genome evolution. An example of the kind of biological inference that can be built upon a well-supported phylogeny is provided by the two endosymbionts included in our set. Wigglesworthia and Buchnera have sometimes been considered as closely related and sometimes not, based on relatively weak phylogenetic evidence provided by the SSU rRNA alone. Our confirmation of their close relationship raises the question of whether their common ancestor was an endosymbiont with a reduced genome or a free-living bacterium (perhaps one with a host-associated lifestyle that promoted formation of intimate symbiosis). Because Buchnera and Wigglesworthia do not share any genes absent from the other species, no particular genes can be implicated as conferring a predisposition to symbiosis, a result that eliminates some hypotheses about how symbiosis originates. Furthermore, although emphasis has previously been placed on the close relationship of Buchnera with E. coli, our results shows that the phylogenetic relationship is equally high with other enterics, such as Yersinia pestis, which indeed shares as many ortholog families with Buchnera as does E. coli. This knowledge of relationships to other genomes allows more accurate reconstruction of ancestral genome content and of the chromosomal deletions and rearrangements occurring during the evolution of reduced symbiotic genomes (Moran and Mira 2001).

One biological interpretation of our findings is that the immediate retention of an acquired gene within a lineage depends upon strong positive selection for its function (Ochman et al. 2000) and that such selection is unlikely if a homologous gene is already present in the recipient genome. An implication, from the perspective of phylogeny reconstruction, is that single-copy homologs with widespread distribution are a source of reliable information for inferring organismal phylogeny. The existence of many other gene families with multiple members per genome or with erratic distributions across the set of genomes (see Figure 1) is consistent with a major role of LGT, gene loss, and gene duplication in the evolution of this bacterial clade. Combined with chromosomal rearrangements, these events are the major sources of genomic, and ultimately ecological, diversification of bacterial groups. By demonstrating the potential to establish robust organismal phylogenies using genome sequence data, our results provide a foundation for examining the rates and frequencies of LGT and other large-scale events in evolving genomes.

## Materials and Methods

### 

#### Data.

The genomes chosen for this study correspond to 13 γ-Proteobacterial taxa that show different degrees of relatedness based on divergence of SSU rRNA and that include two symbionts having undergone large-scale genomic reduction (Shigenobu et al. 2000; Akman et al. 2002). The protein sequences of the 13 complete genomes were retrieved from the GenBank database (Benson et al. 2002). The species used were Escherichia coli K12 (accession number NC_000913; Blattner et al. 1997), Buchnera aphidicola APS (NC_002528; Shigenobu et al. 2000), Haemophilus influenzae Rd (NC_000907; Fleischmann et al. 1995), Pasteurella multocida Pm70 (NC_002663; May et al. 2001), Salmonella typhimurium LT2 (NC_003197; McClelland et al. 2001), Yersinia pestis CO_92 (NC_003143; Parkhill et al. 2000), Yersinia pestis KIM5 P12 (NC_004088; Deng et al. 2002), Vibrio cholerae (NC_002505 for chromosome 1 and NC_002506 for chromosome 2; Heidelberg et al. 2000), Xanthomonas axonopodis pv. citri 306 (NC_003919; da Silva et al. 2002), Xanthomonas campestris (NC_003902; da Silva et al. 2002), Xylella fastidiosa 9a5c (NC_002488; Simpson et al. 2000), Pseudomonas aeruginosa PA01 (NC_002516; Stover et al. 2000), and Wigglesworthia glossinidia brevipalpis (NC_004344; Akman et al. 2002).

To identify genes likely to have been transmitted vertically through the history of the γ-Proteobacteria, we first eliminated proteins annotated as elements of insertion sequences or as bacteriophage sequences, since they are likely to be subject to lateral transfer. Such sequences were present in most genomes but lacking in a few (B. aphidicola, W. brevipalpis, and P. multocida). Table 1 shows the number of proteins that remain in each genome after such elimination.

**Table 1:**
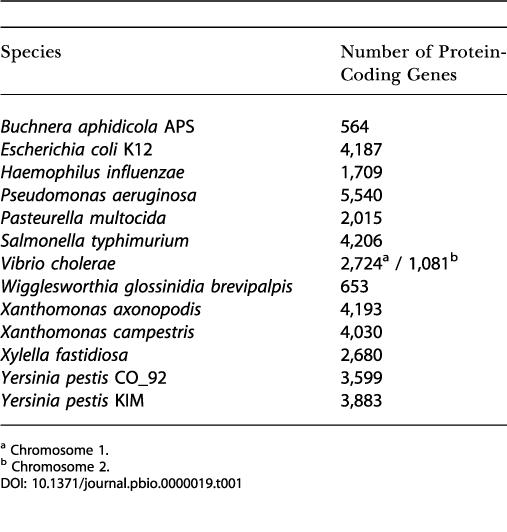
Number of Protein-Coding Genes per Genome after Elimination of the Insertion and Bacteriophage Sequences

^a^ Chromosome 1

^b^ Chromosome 2

#### Construction of the gene families.

**Figure 6:**
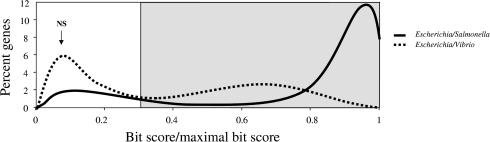
Similarity Levels for Pairwise Comparisons of Genes from Two Representative Genome Pairs Frequency distribution of the ratio (bit score/maximal bit score) in a BLASTP query of the proteins from E. coli on the proteins from the genomes of Salmonella enterica (solid line) and Vibrio cholerae (dashed line). The ratio of 0.3 allows identification of most homologs but excludes probable nonspecific matches (NS).

We applied a stringent criterion for eliminating nonhomologous sequences and paralogous sequences, since both are likely to lead to false conclusions regarding the organismal phylogeny and frequency of LGT. In particular, the criterion of “best reciprocal hits” between sequences for a genome pair can lead to false conclusions of orthology because the resulting gene pairs are not always closest relatives phylogenetically (Koski and Golding 2001). Instead, we used a cutoff for the degree of similarity as reflected in the BLASTP bit scores (Altschul et al. 1997). The bit score is dependent upon the scoring system (substitution matrix and gap costs) employed and takes into account both the degree of similarity and the length of the alignment between the query and the match sequences. We used it to detect homologous genes, described as follows. A bank of all annotated protein sequences of all included species was created. A BLASTP (Altschul et al. 1997) search was performed for all the proteins in each genome against the protein bank. This implies that all proteins were searched against both their resident genome and those from the 12 other species. The match of a given protein against itself gives a maximal bit score. To determine a threshold to group genes into a family, we examined the distribution of the ratio of the bit score to the maximal (self) bit score based on the proteins of E. coli compared against proteins of the 12 genomes (Figure 6). In each case, the distribution showed a clear bimodal pattern with a first peak of low similarity values, which is constant among comparisons and therefore probably represents random matches, and a second peak of higher similarity values, representing true homologous genes. For comparisons of E. coli proteins with those of the most distant species in our set, such as Vibrio, Xanthomonas, Xylella, and Pseudomonas, the separation of the two portions of the distribution occurs at about 30% of the maximal bit score. Thus, in order to apply a stringent criterion for homology, we inferred as homologous genes those presenting a bit score value higher or equal to 30% of the maximal bit score. A protein was included in a family if this criterion was satisfied for at least one member. Our cutoff was chosen to minimize inclusion of nonhomologous sequences within a family; consequently, it may exclude some homologs, especially fast-evolving ones.

After establishing homolog families, we selected the set that contained a single sequence in each represented genome and regarded these as likely orthologs that could give information about the organismal phylogeny and the frequency of LGT affecting orthologs in this bacterial group.

#### Phylogenies.

The alignments for each identified gene family were created using the CLUSTALW 1.8 program (Thompson et al. 1994). We corrected the concatenated proteins alignment by removing ambiguous parts using the SEAVIEW sequence editor (Galtier et al. 1996). The TREE-PUZZLE 5.1 program (Schmidt et al. 2002) was used in order to determine the α parameter from the datasets for the γ-based method for correcting the rate heterogeneity among sites.

We wished to generate a set of reasonable candidate topologies that could be tested against the alignments for individual genes. These topologies were generated based on the consensus of the 205 trees from individual protein families (one method, yielding topology 5 of Figure 2), on the concatenation of all the proteins (over 75,000 amino acids) (two methods, yielding topologies 5 and 6), and on the SSU rRNA (four methods, yielding topologies 1–4). In the case of the reconstruction of the trees based on the SSU rRNA, we used the DNAML module of the PHYLIP package version 3.6 (Felsenstein 2002), which performs ML reconstruction using the γ-based method for correcting the rate heterogeneity among sites; the PHYLO_WIN program (Galtier et al. 1996) using the NJ method with bootstrap and using two different distances, Kimura 2P distance and Galtier and Gouy distance, designed to reduce bias due to base composition (Galtier et al. 1996); and the MEGA program (Kumar et al. 1993) using the NJ method with bootstrap and with the γ-based method for correcting the rate heterogeneity among sites.

We used the PROML module of the PHYLIP package version 3.6 (Felsenstein 2002) to conduct a ML reconstruction using the Jones, Taylor, and Thornton (JTT) model of substitution (Jones et al. 1992) and the γ-based method for correcting the rate heterogeneity among sites, on each of the 205 families of single-copy, orthologous proteins. The consensus of the trees of the 205 protein alignments was obtained using the CONSENSE module of the PHYLIP package version 3.6 (Felsenstein 2002). As there are no missing data, we also concatenated all the proteins and used the PHYLO_WIN program (Galtier et al. 1996), using the NJ method and the PAM distance matrix, and the MEGA program (Kumar et al. 1993), using the NJ method with bootstrap and with the γ-based method for correcting the rate heterogeneity among sites, on the protein concatenation.

For each of the 205 alignments, a comparison of the likelihood of the best topology with the likelihood of the candidate topologies shown in Figure 2 were performed with the SH test (Shimodaira and Hasegawa 1999) implemented in TREE-PUZZLE 5.1 (Schmidt et al. 2002). This test determines whether these potential organismal phylogenies are significantly rejected by the alignment and thus whether an event of LGT must be invoked.

Finally, we used the SH test to determine whether more than one LGT event was required to explain the lack of congruence between the favored topology and two gene alignments that rejected that topology. For each case, we observed which taxon showed the most evident discordance in the topology derived from the exceptional gene alignment. We then removed the corresponding sequence from the alignment and the corresponding taxon from the widely favored topologies. Using an SH test, we determined whether the alignment continued to show significant conflict with the favored topologies.

## Supporting Information

Table S1 Names and Functional Categories of the 205 Genes Used to Reconstruct the Phylogenetical Relationship of γ-Proteobacteria (123 KB DOC).

### Accession Numbers

The GenBank accession numbers discussed in this paper are NC_000907, NC_000913, NC_002488, NC_002505, NC_002506, NC_002516, NC_002528, NC_002663, NC_003143, NC_003197, NC_003902, NC_003919, NC_004088, and NC_004344.

## Abbreviations

BioB: biotin synthase
LGT: lateral gene transfer
ML: maximum likelihood
NJ: neighbor joining
PAM: polyacid modified
SH test: Shimodaira–Hasegawa test
SSU rRNA: small subunit ribosomal RNA.
