# Supplementary material for: From Gene Trees to Organismal Phylogeny in Prokaryotes:The Case of the γ-Proteobacteria
Source: PLoS Biol. 2003 Sep 15;1(1):e19. doi: 10.1371/journal.pbio.0000019 (PMC193605; doi:10.1371/journal.pbio.0000019)
Supplement: Table S1 — (123 KB DOC). [file pbio.0000019.st001.doc]

Supplementary table: names and functional categories of the 205 genes used to reconstruct the phylogenetical relationship of gamma-proteobacteria.

| name | Role | Function | decription in B.aphidicola |
| --- | --- | --- | --- |
| aroK | Amino acid biosynthesis | Aromatic amino acid family | shikimate kinase I |
| dapA | Amino acid biosynthesis | Aspartate family (lysine) | dihydrodipicolinate synthase |
| dapB | Amino acid biosynthesis | Aspartate family (lysine) | dihydrodipicolinate reductase |
| dapE | Amino acid biosynthesis | Aspartate family (lysine) | succinyl-diaminopimelate desuccinylase |
| dapF | Amino acid biosynthesis | Aspartate family (lysine) | diaminopimelate epimerase |
| bioA | Biosynthesis of cofactors, prosthetic groups, and carriers | Biotin | adenosylmethionine-8-amino-7-oxononanoate aminotransferase |
| bioB | Biosynthesis of cofactors, prosthetic groups, and carriers | Biotin | biotin synthetase |
| folA | Biosynthesis of cofactors, prosthetic groups, and carriers | Folic acid | dihydrofolate reductase |
| folC | Biosynthesis of cofactors, prosthetic groups, and carriers | Folic acid | folylpolyglutamate synthase / dihydrofolate synthase |
| folD | Biosynthesis of cofactors, prosthetic groups, and carriers | Folic acid | methylenetetrahydrofolate dehydrogenase / methenyltetrahydrofolate cyclohydrolase |
| lipA | Biosynthesis of cofactors, prosthetic groups, and carriers | Lipoate | lipoic acid synthetase |
| lipB | Biosynthesis of cofactors, prosthetic groups, and carriers | Lipoate | lipoate-protein ligase B |
| ispA | Biosynthesis of cofactors, prosthetic groups, and carriers | Menaquinone and ubiquinone | geranyltranstransferase |
| dxr | Biosynthesis of cofactors, prosthetic groups, and carriers | Other | 1-deoxy-D-xylulose 5-phosphate reductoisomerase |
| dxs | Biosynthesis of cofactors, prosthetic groups, and carriers | Other | dxs protein |
| metK | Biosynthesis of cofactors, prosthetic groups, and carriers | Other | S-adenosylmethionine synthetase |
| uppS | Biosynthesis of cofactors, prosthetic groups, and carriers | Other | undecaprenyl pyrophosphate synthetase |
| kdtB | Biosynthesis of cofactors, prosthetic groups, and carriers | Pantothenate and coenzyme A | lipopolysaccharide core biosynthesis protein kdtB |
| ribA | Biosynthesis of cofactors, prosthetic groups, and carriers | Riboflavin, FMN, and FAD | GTP cyclohydrolase II |
| ribE | Biosynthesis of cofactors, prosthetic groups, and carriers | Riboflavin, FMN, and FAD | riboflavin synthase alpha chain |
| ribF | Biosynthesis of cofactors, prosthetic groups, and carriers | Riboflavin, FMN, and FAD | riboflavin kinase / FMN adenylyltransferase |
| ribH | Biosynthesis of cofactors, prosthetic groups, and carriers | Riboflavin, FMN, and FAD | 6,7-dimethyl-8-ribityllumazine synthase |
| glmU | Cell envelope | Biosynthesis of murein sacculus and peptidoglycan | UDP-N-acetylglucosamine pyrophosphorylase |
| mraY | Cell envelope | Biosynthesis of murein sacculus and peptidoglycan | phospho-N-acetylmuramoyl-pentapeptide-transferase |
| mrcB | Cell envelope | Biosynthesis of murein sacculus and peptidoglycan | penicillin-binding protein 1b |
| murA | Cell envelope | Biosynthesis of murein sacculus and peptidoglycan | UDP-N-acetylglucosamine 1-carboxyvinyltransferase |
| murB | Cell envelope | Biosynthesis of murein sacculus and peptidoglycan | UDP-N-acetylenolpyruvoylglucosamine reductase |
| murC | Cell envelope | Biosynthesis of murein sacculus and peptidoglycan | UDP-N-acetylmuramate-alanine ligase |
| murE | Cell envelope | Biosynthesis of murein sacculus and peptidoglycan | UDP-N-acetylmuramoylalanyl-D-glutamate-2, 6-diaminopimelate ligase |
| murF | Cell envelope | Biosynthesis of murein sacculus and peptidoglycan | UDP-N-acetylmuramoylalanyl-D-glutamyl-2, 6-diaminopimelate-D-alanyl-D-alanyl ligase |
| murG | Cell envelope | Biosynthesis of murein sacculus and peptidoglycan | UDP-N-acetylglucosamine-N-acetylmuramyl-(pentape ptide) pyrophosphoryl-undecaprenol N-acetylglucosamine transferase |
| yidC | Cell envelope | Other | 60 kD inner-membrane protein |
| hslU | Cellular processes | Adaptations to atypical conditions | heat shock protein hslU |
| htpX | Cellular processes | Adaptations to atypical conditions | heat shock protein htpX |
| ftsA | Cellular processes | Cell division | cell division protein ftsA |
| ftsJ | Cellular processes | Cell division | cell division protein ftsJ |
| ftsY | Cellular processes | Cell division | cell division protein ftsY |
| ftsZ | Cellular processes | Cell division | cell division protein ftsZ |
| thdF | Cellular processes | Detoxification | thiophene and furan oxidation protein thdF |
| ksgA | Cellular processes | Toxin production and resistance | dimethyladenosine transferase |
| lytB | Cellular processes | Toxin production and resistance | lytB protein |
| glmS | Central intermediary metabolism | Amino sugars | D-fructose-6-phosphate amidotransferase |
| dnaB | DNA metabolism | DNA replication, recombination, and repair | replicative DNA helicase |
| dnaE | DNA metabolism | DNA replication, recombination, and repair | DNA polymerase III alpha chain |
| dnaG | DNA metabolism | DNA replication, recombination, and repair | DNA primase |
| dnaN | DNA metabolism | DNA replication, recombination, and repair | DNA polymerase III beta chain |
| dnaQ | DNA metabolism | DNA replication, recombination, and repair | DNA polymerase III epsilon chain |
| dnaX | DNA metabolism | DNA replication, recombination, and repair | DNA polymerase III subunits gamma and tau |
| gidA | DNA metabolism | DNA replication, recombination, and repair | glucose inhibited division protein A |
| gyrA | DNA metabolism | DNA replication, recombination, and repair | DNA gyrase subunit A |
| lig | DNA metabolism | DNA replication, recombination, and repair | DNA ligase (NAD+) |
| mfd | DNA metabolism | DNA replication, recombination, and repair | transcription-repair coupling factor |
| nth | DNA metabolism | DNA replication, recombination, and repair | endonuclease III |
| polA | DNA metabolism | DNA replication, recombination, and repair | DNA polymerase I |
| hemK | DNA metabolism | Restriction/modification | hemK protein |
| atpA | Energy metabolism | ATP-proton motive force interconversion | ATP synthase alpha chain |
| atpB | Energy metabolism | ATP-proton motive force interconversion | ATP synthase A chain |
| atpC | Energy metabolism | ATP-proton motive force interconversion | ATP synthase epsilon chain |
| atpD | Energy metabolism | ATP-proton motive force interconversion | ATP synthase beta chain |
| atpE | Energy metabolism | ATP-proton motive force interconversion | ATP synthase C chain |
| atpF | Energy metabolism | ATP-proton motive force interconversion | ATP synthase B chain |
| atpG | Energy metabolism | ATP-proton motive force interconversion | ATP synthase gamma chain |
| eno | Energy metabolism | Glycolysis/gluconeogenesis | enolase |
| pgi | Energy metabolism | Glycolysis/gluconeogenesis | glucose-6-phosphate isomerase |
| pgk | Energy metabolism | Glycolysis/gluconeogenesis | phosphoglycerate kinase |
| tpiA | Energy metabolism | Glycolysis/gluconeogenesis | triosephosphate isomerase |
| gloB | Energy metabolism | Other | probable hydroxyacylglutathione hydrolase |
| aceE | Energy metabolism | Pyruvate dehydrogenase | pyruvate dehydrogenase e1 component |
| aceF | Energy metabolism | Pyruvate dehydrogenase | dihydrolipoamide acetyltransferase |
| sucA | Energy metabolism | TCA cycle | 2-oxoglutarate dehydrogenase e1 component |
| sucB | Energy metabolism | TCA cycle | dihydrolipoamide succinyltransferase component (E2) of 2-oxoglutarate dehydrogenase complex |
| clpP | Protein fate | Degradation of proteins, peptides, and glycopeptides | ATP-dependent clp protease ATP-binding subunit clpX |
| hslV | Protein fate | Degradation of proteins, peptides, and glycopeptides | heat shock protein hslV |
| pepA | Protein fate | Degradation of proteins, peptides, and glycopeptides | aminopeptidase A/I |
| ygjD | Protein fate | Degradation of proteins, peptides, and glycopeptides | O-sialoglycoprotein endopeptidase |
| ffh | Protein fate | Protein and peptide secretion and trafficking | signal recognition particle protein |
| lspA | Protein fate | Protein and peptide secretion and trafficking | lipoprotein signal peptidase |
| secE | Protein fate | Protein and peptide secretion and trafficking | preprotein translocase secE subunit |
| secY | Protein fate | Protein and peptide secretion and trafficking | preprotein translocase secY subunit |
| dnaJ | Protein fate | Protein folding and stabilization | dnaJ protein |
| htpG | Protein fate | Protein folding and stabilization | heat shock protein htpG |
| lgt | Protein fate | Protein modification and repair | prolipoprotein diacylglyceryl transferase |
| pth | Protein synthesis | Other | peptidyl-tRNA hydrolase |
| smpB | Protein synthesis | Other | small protein B |
| rplA | Protein synthesis | Ribosomal proteins: synthesis and modification | 50S ribosomal protein L1 |
| rplB | Protein synthesis | Ribosomal proteins: synthesis and modification | 50S ribosomal protein L2 |
| rplC | Protein synthesis | Ribosomal proteins: synthesis and modification | 50S ribosomal protein L3 |
| rplD | Protein synthesis | Ribosomal proteins: synthesis and modification | 50S ribosomal protein L4 |
| rplE | Protein synthesis | Ribosomal proteins: synthesis and modification | 50S ribosomal protein L5 |
| rplF | Protein synthesis | Ribosomal proteins: synthesis and modification | 50S ribosomal protein L6 |
| rplI | Protein synthesis | Ribosomal proteins: synthesis and modification | 50S ribosomal protein L9 |
| rplJ | Protein synthesis | Ribosomal proteins: synthesis and modification | 50S ribosomal protein L10 |
| rplK | Protein synthesis | Ribosomal proteins: synthesis and modification | 50S ribosomal protein L11 |
| rplL | Protein synthesis | Ribosomal proteins: synthesis and modification | 50S ribosomal protein L7/L12 |
| rplM | Protein synthesis | Ribosomal proteins: synthesis and modification | 50S ribosomal protein L13 |
| rplN | Protein synthesis | Ribosomal proteins: synthesis and modification | 50S ribosomal protein L14 |
| rplO | Protein synthesis | Ribosomal proteins: synthesis and modification | 50S ribosomal protein L15 |
| rplP | Protein synthesis | Ribosomal proteins: synthesis and modification | 50S ribosomal protein L16 |
| rplQ | Protein synthesis | Ribosomal proteins: synthesis and modification | 50S ribosomal protein L17 |
| rplR | Protein synthesis | Ribosomal proteins: synthesis and modification | 50S ribosomal protein L18 |
| rplS | Protein synthesis | Ribosomal proteins: synthesis and modification | 50S ribosomal protein L19 |
| rplT | Protein synthesis | Ribosomal proteins: synthesis and modification | 50S ribosomal protein L20 |
| rplU | Protein synthesis | Ribosomal proteins: synthesis and modification | 50S ribosomal protein L21 |
| rplV | Protein synthesis | Ribosomal proteins: synthesis and modification | 50S ribosomal protein L22 |
| rplW | Protein synthesis | Ribosomal proteins: synthesis and modification | 50S ribosomal protein L23 |
| rplX | Protein synthesis | Ribosomal proteins: synthesis and modification | 50S ribosomal protein L24 |
| rplY | Protein synthesis | Ribosomal proteins: synthesis and modification | 50S ribosomal protein L25 |
| rpmA | Protein synthesis | Ribosomal proteins: synthesis and modification | 50S ribosomal protein L27 |
| rpmC | Protein synthesis | Ribosomal proteins: synthesis and modification | 50S ribosomal protein L29 |
| rpmD | Protein synthesis | Ribosomal proteins: synthesis and modification | 50S ribosomal protein L30 |
| rpmF | Protein synthesis | Ribosomal proteins: synthesis and modification | 50S ribosomal protein L32 |
| rpmG | Protein synthesis | Ribosomal proteins: synthesis and modification | 50S ribosomal protein L33 |
| rpmH | Protein synthesis | Ribosomal proteins: synthesis and modification | 50S ribosomal protein L34 |
| rpsA | Protein synthesis | Ribosomal proteins: synthesis and modification | 30S ribosomal protein S1 |
| rpsB | Protein synthesis | Ribosomal proteins: synthesis and modification | 30S ribosomal protein S2 |
| rpsC | Protein synthesis | Ribosomal proteins: synthesis and modification | 30S ribosomal protein S3 |
| rpsD | Protein synthesis | Ribosomal proteins: synthesis and modification | 30S ribosomal protein S4 |
| rpsE | Protein synthesis | Ribosomal proteins: synthesis and modification | 30S ribosomal protein S5 |
| rpsF | Protein synthesis | Ribosomal proteins: synthesis and modification | 30S ribosomal protein S6 |
| rpsG | Protein synthesis | Ribosomal proteins: synthesis and modification | 30S ribosomal protein S7 |
| rpsH | Protein synthesis | Ribosomal proteins: synthesis and modification | 30S ribosomal protein S8 |
| rpsI | Protein synthesis | Ribosomal proteins: synthesis and modification | 30S ribosomal protein S9 |
| rpsJ | Protein synthesis | Ribosomal proteins: synthesis and modification | 30S ribosomal protein S10 |
| rpsK | Protein synthesis | Ribosomal proteins: synthesis and modification | 30S ribosomal protein S11 |
| rpsL | Protein synthesis | Ribosomal proteins: synthesis and modification | 30S ribosomal protein S12 |
| rpsM | Protein synthesis | Ribosomal proteins: synthesis and modification | 30S ribosomal protein S13 |
| rpsN | Protein synthesis | Ribosomal proteins: synthesis and modification | 30S ribosomal protein S14 |
| rpsP | Protein synthesis | Ribosomal proteins: synthesis and modification | 30S ribosomal protein S16 |
| rpsR | Protein synthesis | Ribosomal proteins: synthesis and modification | 30S ribosomal protein S18 |
| rpsT | Protein synthesis | Ribosomal proteins: synthesis and modification | 30S ribosomal protein S20 |
| rpsU | Protein synthesis | Ribosomal proteins: synthesis and modification | 30S ribosomal protein S21 |
| frr | Protein synthesis | Translation factors | ribosome recycling factor |
| infA | Protein synthesis | Translation factors | translation initiation factor IF-1 |
| infB | Protein synthesis | Translation factors | translation initiation factor IF-2 |
| infC | Protein synthesis | Translation factors | translation initiation factor IF-3 |
| tsf | Protein synthesis | Translation factors | elongation factor Ts |
| alaS | Protein synthesis | tRNA aminoacylation | alanyl-tRNA synthetase |
| aspS | Protein synthesis | tRNA aminoacylation | aspartyl-tRNA synthetase |
| cysS | Protein synthesis | tRNA aminoacylation | cysteinyl-tRNA synthetase |
| fmt | Protein synthesis | tRNA aminoacylation | methionyl-tRNA formyltransferase |
| glnS | Protein synthesis | tRNA aminoacylation | glutaminyl-tRNA synthetase |
| gltX | Protein synthesis | tRNA aminoacylation | glutamyl-tRNA synthetase |
| glyQ | Protein synthesis | tRNA aminoacylation | glycyl-tRNA synthetase alpha chain |
| glyS | Protein synthesis | tRNA aminoacylation | glycyl-tRNA synthetase beta chain |
| ileS | Protein synthesis | tRNA aminoacylation | isoleucyl-tRNA synthetase |
| pheS | Protein synthesis | tRNA aminoacylation | phenylalanyl-tRNA synthetase alpha chain |
| pheT | Protein synthesis | tRNA aminoacylation | phenylalanyl-tRNA synthetase beta chain |
| proS | Protein synthesis | tRNA aminoacylation | prolyl-tRNA synthetase |
| serS | Protein synthesis | tRNA aminoacylation | seryl-tRNA synthetase |
| thrS | Protein synthesis | tRNA aminoacylation | threonyl-tRNA synthetase |
| valS | Protein synthesis | tRNA aminoacylation | valyl-tRNA synthetase |
| miaA | Protein synthesis | tRNA and rRNA base modification | tRNA delta(2)-isopentenylpyrophosphate transferase |
| truA | Protein synthesis | tRNA and rRNA base modification | pseudouridylate synthase I |
| truB | Protein synthesis | tRNA and rRNA base modification | tRNA pseudouridine 55 synthase |
| adk | Purines, pyrimidines, nucleosides, and nucleotides | Nucleotide and nucleoside interconversions | adenylate kinase |
| gmk | Purines, pyrimidines, nucleosides, and nucleotides | Nucleotide and nucleoside interconversions | guanylate kinase |
| apaH | Purines, pyrimidines, nucleosides, and nucleotides | Other | bis(5'-nucleosyl)-tetraphosphatase (symmetrical) |
| prsA | Purines, pyrimidines, nucleosides, and nucleotides | Purine ribonucleotide biosynthesis | ribose-phosphate pyrophosphokinase |
| purA | Purines, pyrimidines, nucleosides, and nucleotides | Purine ribonucleotide biosynthesis | adenylosuccinate synthetase |
| purB | Purines, pyrimidines, nucleosides, and nucleotides | Purine ribonucleotide biosynthesis | adenylosuccinate lyase |
| purH | Purines, pyrimidines, nucleosides, and nucleotides | Purine ribonucleotide biosynthesis | phosphoribosylaminoimidazolecarboxamide formyltransferase / IMP cyclohydrolase |
| pyrD | Purines, pyrimidines, nucleosides, and nucleotides | Pyrimidine ribonucleotide biosynthesis | dihydroorotate dehydrogenase |
| pyrE | Purines, pyrimidines, nucleosides, and nucleotides | Pyrimidine ribonucleotide biosynthesis | orotate phosphoribosyltransferase |
| pyrF | Purines, pyrimidines, nucleosides, and nucleotides | Pyrimidine ribonucleotide biosynthesis | orotidine 5'-phosphate decarboxylase |
| pyrG | Purines, pyrimidines, nucleosides, and nucleotides | Pyrimidine ribonucleotide biosynthesis | CTP synthase |
| orn | Transcription | Degradation of RNA | oligoribonuclease |
| pnp | Transcription | Degradation of RNA | polyribonucleotide nucleotidyltransferase |
| rpoA | Transcription | DNA-dependent RNA polymerase | DNA-directed RNA polymerase alpha chain |
| rpoB | Transcription | DNA-dependent RNA polymerase | DNA-directed RNA polymerase beta chain |
| rpoC | Transcription | DNA-dependent RNA polymerase | DNA-directed RNA polymerase beta' chain |
| cca | Transcription | RNA processing | tRNA nucleotidyltransferase |
| rbfA | Transcription | RNA processing | ribosome-binding factor A |
| rimM | Transcription | RNA processing | 16s rRNA processing protein rimm |
| rnc | Transcription | RNA processing | ribonuclease III |
| rnt | Transcription | RNA processing | ribonuclease T |
| trmU | Transcription | RNA processing | tRNA (5-methylaminomethyl-2-thiouridylate)-methy ltransferase |
| nusA | Transcription | Transcription factors | N utilization substance protein A |
| nusG | Transcription | Transcription factors | transcription antitermination protein nusG |
| rho | Transcription | Transcription factors | transcription termination factor rho |
| rpoH | Transcription | Transcription factors | RNA polymerase sigma-32 factor |
| engA | Unknown function | General | hypothetical GTP-binding protein |
| lepA | Unknown function | General | GTP-binding protein lepA |
| mviN | Unknown function | General | virulence factor mviN homolog |
| suhB | Unknown function | General | extragenic suppressor protein suhB |
| yabC | Unknown function | General | hypothetical protein |
| yacE | Unknown function | General | hypothetical protein |
| yaeT | Unknown function | General | hypothetical protein |
| yajC | Unknown function | General | hypothetical protein |
| ybeY | Unknown function | General | hypothetical protein |
| ychB | Unknown function | General | hypothetical protein |
| ychF | Unknown function | General | GTP-binding protein |
| yfgB | Unknown function | General | hypothetical protein |
| yfhC | Unknown function | General | hypothetical protein yfhC |
| ygbB | Unknown function | General | hypothetical protein |
| yggJ | Unknown function | General | hypothetical protein |
| yggS | Unknown function | General | hypothetical protein |
| yggX | Unknown function | General | hypothetical protein |
| yhbZ | Unknown function | General | hypothetical 43.3 kD GTP-binding protein in dacB-rpmA intergenic region (F390) |
| yhgI | Unknown function | General | hypothetical protein |
| yhhF | Unknown function | General | hypothetical protein |
| yibN | Unknown function | General | hypothetical protein |
| yihA | Unknown function | General | hypothetical GTP-binding protein |
| yqgF | Unknown function | General | hypothetical protein |
| yraL | Unknown function | General | hypotheical protein |
| yrdC | Unknown function | General | hypothetical protein |
